# Phytotherapy in Urolithiasis: An Updated Overview of Current Knowledge

**DOI:** 10.3390/jcm14092885

**Published:** 2025-04-22

**Authors:** Wilbert F. Mutomba, Evangelos N. Symeonidis, Ioannis Mykoniatis, Lazaros Tzelves, Arman Tsaturyan, Patrick Juliebo-Jones, Theodoros Tokas, Petros Sountoulides

**Affiliations:** 1 First Department of Urology, Faculty of Medicine, School of Health Sciences, Aristotle University of Thessaloniki, 541 24 Thessaloniki, Greece; w_mutomba@yahoo.com (W.F.M.); g_mikoniatis@hotmail.com (I.M.); 2 Second Department of Urology, Interbalkan Medical Center, 555 35 Thessaloniki, Greece; evansimeonidis@gmail.com; 3 European Association of Urology-Young Academic Urologists (EAU-YAU), Urolithiasis and Endourology Working Group, NL-6803 AA Arnhem, The Netherlands; lazarostzelves@gmail.com (L.T.); tsaturyanarman@yahoo.com (A.T.);; 4 Second Department of Urology, National and Kapodistrian University of Athens, Sismanogleio General Hospital, 151 26 Marousi, Greece; 5 Department of Urology, Erebouni Medical Center, Yerevan 0087, Armenia; 6 Department of Urology, Haukeland University Hospital, 5009 Bergen, Norway; 7 Department of Urology, Medical School, University General Hospital of Heraklion, University of Crete, 700 13 Heraklion, Greece; ttokas@yahoo.com

**Keywords:** herbal medicines, phytotherapy, kidney stones, urinary calculi, urolithiasis

## Abstract

Urolithiasis is one of the most burdensome urinary tract conditions with a prevalence ranging from 1% to 20%. Approximately half of the patients experience a recurrence, while 10% face multiple stone episodes. Long before the advent of surgical treatment options, herbal therapy, or phytotherapy, had been used for both the prevention and management of urolithiasis. Recently, interest in phytotherapy has been rekindled due to the limitations associated with modern urolithiasis treatment, the limited options for conventional medical therapy, and the added cost of interventions for stones. While research on phytotherapy is still limited, it is ongoing and is yielding promising results. In order to capture the current trend in phytotherapy for urolithiasis, we performed a narrative review from data collected and synthesized from electronic databases, with a specific focus on randomized human studies. Our analysis revealed that the use of various herbal medicines and phytotherapy, either as mixtures or as sole plant extracts, in urolithiasis is on the rise and is mainly utilized as complementary therapy to conventional treatment. Although most studies demonstrate the effectiveness of phytotherapy in reducing stone size and facilitating stone expulsion, several questions regarding specific dosages, mechanisms of action, drug interactions, treatment duration, and types of stones that respond to phytotherapy remain unanswered. This review aims to summarize the current knowledge surrounding the role of phytotherapy in urolithiasis and to determine its role as a primary or complementary treatment alongside traditional treatment options. Ultimately, further research is essential to clarify the abovementioned unresolved issues, overcome the existing challenges, and optimize the best possible phytotherapy strategies and timing for dissolving specific types of stones with these regimens.

## 1. Introduction

Urolithiasis, also known as urinary stone disease, is the presence of stones in the lower (bladder, urethra) or upper (kidneys, ureter) urinary tract. According to extensive epidemiological studies, the prevalence of urolithiasis in the general population in economically developed countries ranges from 1% to 20% [1]. The prevalence of urolithiasis varies according to geographical area and is related to sex, age, diet, and climate factors [2]. One in two stone formers will have at least another stone episode during their lifetime, while 10% of patients will suffer multiple stone episodes [3,4]. Nearly half of the stone formers will have another episode within 10 years [5]. Diet plays an important role in urolithiasis, with those following a plant-based diet presenting with lower risk of stone formation [6]. Modification of diet can help reduce stone recurrence rates, especially for patients who would have undergone a metabolic stone work-up [7].

**Figure 1:**
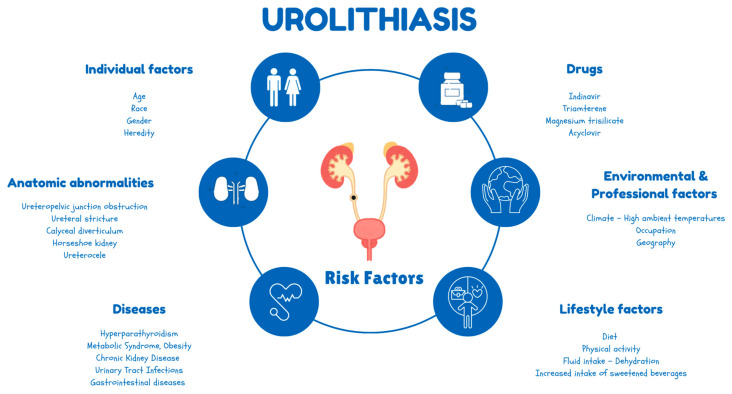
Schematic presentation of the most common causes and predisposing risk factors of urolithiasis.

Urinary stone disease is the third most common pathological condition affecting the urinary tract, after urinary tract infections and prostate conditions [8]. The size, site, and number (single or multiple) of stone(s), distinct urinary system anatomy, comorbidities, age, and individual performance status are essential for treatment planning [9]. Urolithiasis is a disorder with significant socioeconomic implications, influencing the quality of life. Urinary stones are a recurrent disorder of multifactorial origin, all of which need to be assessed, and their understanding should be used to influence management [10]. A schematic presentation of the most common causes and predisposing risk factors of urolithiasis is depicted in Figure 1.

**Figure 2:**
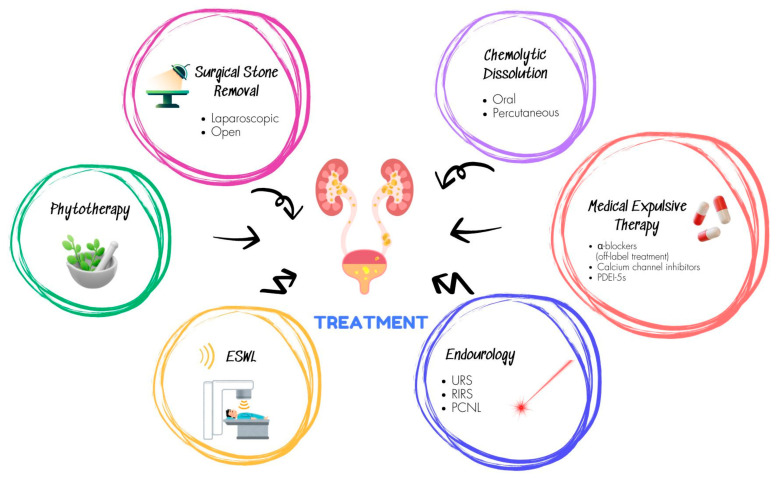
Overview of the available treatment options for urolithiasis.

The treatment of urolithiasis is a matter of socioeconomic concern. Medical Expulsive Therapy (MET), Shockwave Lithotripsy (SWL), Ureteroscopy (URS), Retrograde Intrarenal Surgery (RIRS), and Percutaneous Nephrolithotomy (PCNL) are the available options depending on stone size and location, stone burden, comorbidities, and patient preferences [11]. Stone factors, anatomy, patient factors, and the surgeon’s preference determine the optimal treatment selection [12]. There has been a shift in the management of urinary stone disease from inpatient to outpatient setting, and hospitalization time has decreased due to minimally invasive treatment modalities; costs, however, continue to rise, likely due to the increasing prevalence of urinary stone disease [13,14]. Treatment of urolithiasis remains a challenge for conventional medicine. Figure 2 shows an overview of the available treatment options for urolithiasis.

Phytotherapy has been used since time immemorial in traditional practices and has gained traction lately, mainly due to the limitations of conventional medical therapy [15]. The mechanisms of action for certain herbal supplements have yet to be fully elucidated [16]. Current evidence suggests multiple ways of actions including but not limited to increasing diuresis, enhancing citraturia, decreasing calcinuria and oxaluria, inhibiting of nucleation and aggregation of crystals, stone dissolution, and elevating the glycosaminoglycan level [17,18,19]. There is evidence supporting the use of phytotherapy in urolithiasis and UTI management as alternative or adjunct therapy [20,21,22,23]. Some studies have shown that there is also evidence that plant flavonoids inhibit stone formation both in vitro and in vivo [24]. There are many phytotherapy/food supplement products that renal lithiasis patients use over the counter. However, there are limited clinical studies on manufacturers’ claims and limited information on side effect profiles despite the high rating by some patients [25,26]. To date, the effects of these food supplements or phytotherapy are still unknown or understudied in humans; the unavailability of evidence must not be taken to imply the absence of potential harm [27].

This review aims to explore the role of phytotherapy in urolithiasis and identify gaps in knowledge relevant to using such compounds to prevent recurrent episodes and diminish stone formation. The ultimate goal is to increase awareness among urologists and better prepare future urologists treating de novo or recurrent stone formers.

## 2. Materials and Methods

**Figure 3:**
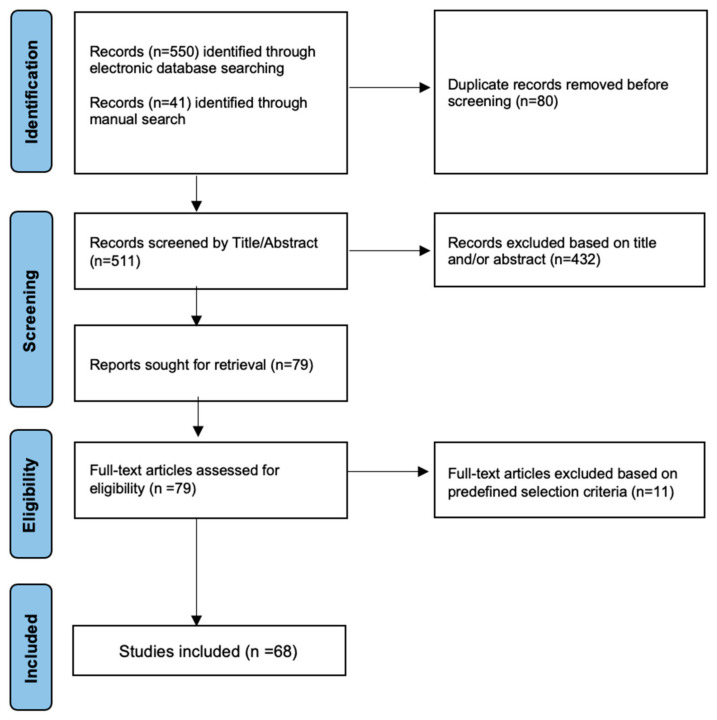
Flow chart of the included studies in the narrative synthesis.

The electronic databases of PubMed and Scopus were searched for English-written articles on phytotherapy and herbal remedies in urolithiasis published from inception through November 2024. The keywords used were “urolithiasis”, “stone disease”, “herbal”, and “phytotherapy”. These search terms were combined with Boolean operators (AND, OR) to refine the search better. One author (WFM) performed the initial search, which resulted in the retrieval of 550 articles. Subsequently, the references of the studies were screened to identify additional articles, thereby expanding the collected data by 41 articles. Following duplicate removal, 511 articles were scrutinized based on their title and abstracts. The search was performed iteratively throughout the review process to capture up-to-date information. Articles of any design, except for case reports, questionnaires, comments, and editorials, were evaluated, and those eligible underwent full-text screening. Special emphasis was placed on research that reports findings from randomized controlled trials, particularly those published within the last two decades. In addition, studies reporting on animals, irrelevant topics or outdated content, outside the treatment scope, or not reporting the use of phytotherapy for urolithiasis were unanimously excluded. In cases of ambiguity, a second author (ENS) followed the same search strategy and reviewed the relevance of the selected manuscripts. Ultimately, a total of 68 articles were included in the final analysis. The search strategy is highlighted through a flow diagram in Figure 3.

## 3. Phytotherapy: Current Evidence from Clinical Trials

A randomized, controlled, open-label pilot study comparing the efficacy of *Celosia argentea* (Cock’s Comb) seeds (titled Sitivaraka) with potassium citrate was conducted. An ultrasound scan was used to determine Sitivaraka’s effects on 44 participants with an average stone size of 8 mm. Twenty-one participants received Sitivaraka thrice daily, while the 23 participants in the control group received potassium citrate for 6 months. The Sitivaraka group showed a decrease in Parathyroid Hormone (PTH) and a reduction in stone size at 3 and 6 months, while the potassium citrate group did not have significant reductions. The results, despite being on a small sample, showed the effectiveness of Cock’s Comb seeds on urinary stones compared with potassium citrate after a 6-month treatment [28].

*Phyllanthus niruri* (Chanca Piedra), or “stone breaker”, is a tropical herb growing in tropical and subtropical areas like the rainforests of South America. Its tea has been used to treat many ailments, including urinary stone disease, in countries like Brazil [29]. A study was conducted investigating the effect of *P. niruri* on the urinary metabolic parameters of 56 patients with kidney stones < 10 mm. No significant anthropometric and serum measurements were noticed in the results. Still, there was an increase in urinary potassium, magnesium/creatinine ratio, and potassium/creatinine ratio, accompanied by a decrease in stone size. Urinary oxalate was reduced in a group of patients with hyperoxaluria, while uric acid decreased in patients with hyperuricosuria. These results demonstrate the safety and effectiveness of “stone breaker” in the elimination of urinary stones and the reduction in stone size [30]. A review suggested the need for RCTs to determine the therapeutic properties of *P. niruri*, but the preventive effect in stone formation or elimination was elucidated [31]. In a comprehensive review of the literature, *Phyllanthus niruri* was shown to interfere with calcium oxalate crystallization while at the same time reducing hyperoxaluria and hyperuricosuria [32].

In another single-arm study, 48 participants were recruited to take 225 mg capsules of *P. niruri* dried leaf extract mixed with 152 mg magnesium stearate and 2 mg pyridoxine hydrochloride (vitamin B6) for 3 months. A non-contrast CT scan was used to determine stone parameters before and after intervention, and recruited participants had a maximum stone diameter of 15 mm before intervention in this study. Less than 3 mm of upper- and mid-calyx stones were expelled, and 3–4 mm stones reduced in size, but the bigger stones were not affected. The study recommended a prolonged treatment duration to increase the intervention’s effectiveness [33]. A systematic review and meta-analysis on *P. niruri* showed limited clinical evidence supporting its efficacy in stone size reduction [34].

Black seed (*Nigella sativa*) has been a Middle Eastern medicinal herb since time immemorial and has been considered effective in treating kidney stones when mixed with honey and water. Black seed’s effects on stones were investigated in a randomized, double-blind, placebo-controlled clinical trial involving 60 participants, 30 in each group. The participants had at least a 5 mm stone, and they took two tablets of 500 mg encapsulated black seed powder daily for 10 weeks in the interventional group and two tablets of placebo in the control group. The results, assessed by a pre- and post-intervention ultrasound scan, showed a 44.4% stone expulsion and a 51.8% stone reduction compared with a 15.3% expulsion and an 11.5% size reduction in the control group. Of note in the results is a 15.3% increase in stone size in the control group, signifying black seed’s effectiveness on urinary stone treatment [35].

Jalal et al. investigated the antiurolithic effects of *Phaseolus vulgaris*, the common beans grown and eaten worldwide in their RCT, placebo-controlled, involving 60 patients with stones less than or equal to 10 mm. Urinary volume and potassium significantly increased after 6 weeks of treatment in the intervention group, and urinary calcium, oxalate, and uric acid significantly decreased compared with the placebo group, while there was a slight increase in urine pH and magnesium. A significant decrease in the number of stones and a reduction in stone sizes on ultrasound scan in the treatment group were seen compared with the placebo arm, indicating the effectiveness of common beans on stone treatment [36].

Erickson et al. investigated the effects of Cystone^®^ tablets on urinary composition and stone formation in a year-long study. Cystone^®^ tablets are one of the traditional Indian Ayurvedic treatments for stones. It was investigated in a first-phase randomized double-blinded 12-week cross-over study for its alterations in urinary supersaturation (6 weeks), and there was no statistically significant effect of Cystone^®^ on urinary composition. The open-label 1-year-duration second phase aimed to determine the effectiveness on stone burden using a CT scan. Again, no statistically significant effect was recorded, leading to the study deduction not supporting the efficacy of Cystone^®^ on renal stones [37]. The study was limited by its very small sample size, as it included 10 patients, all of whom were also recurrent stone formers [38].

Differently, an open-label study on 65 patients with stones between 5 and 12 mm taking two Cystone Forte tablets twice a day for 3 months showed significant stone expulsion, reduction in stone size, and general amelioration of clinical symptoms. This study concluded that Cystone is safe and effective in managing renal stones [39]. Another prospective randomized placebo-controlled study by Patki et al. concluded that Cystone^®^ effectively managed ureteric stones by reducing stone size, improving passage rate, and reducing pain [40]. A systemic review that cited two RCTs [40,41] deducted that Cystone^®^ was effective compared with placebo in lowering renal stone size and stone clearance rates [42].

The Wu-Ling-San (WLS) formula is a traditional Chinese medicine for pain and urinary stone disease [43]. The WLS formula is a mixture made up of the following five herbs: *Rhizoma alismatis*, *Poria cocos Wolf*, *Polyporus umbellatus Fries*, *Rhizoma Atractylodis Macrocephalae*, and *Ramulus Cinnamomi Cassiae* mixed in a weight ratio of 4:3:3:3:2. In a 1-month duration RCT of 28 recurrent stone formers with proven calcium oxalate urinary stones, half taking 2 g WLS three times a day and the other half taking placebo three times a day, the 24-hr urine output, which was used as a measure, increased in the treatment group compared with placebo. WLS was proven to be safe and to increase urine output [44]. On the contrary, in a study investigating the clinical efficacy of WLS for the prevention of recurrent nephrolithiasis, its long-term use did not have a preventive effect on the need for urolithiasis surgical treatment [43]. These two studies did not confirm the effectiveness of WLS.

Mujumdar et al. investigated the safety and efficacy of Subap in a 28-week randomized, double-blind, placebo-controlled study. Subap is an herbal formulation by mixing the dried stem bark of *C. nurvala Buch-ham*, the stem and roots of *Musa* × *paradisiaca Linn*, the whole plant of *A. aspera* Linn, and the seeds of *H. vulgare* Linn. Asymptomatic patients with stone sizes between 4 and 9 mm were enrolled, and the treatment group received Subap, which was compared with placebo. Results showed a statistically significant increased stone expulsion rate, reduced stone density, and reduced stone surface area for the treatment group compared with placebo [45].

The antiurolithiatic effects of another mixture of five plant extracts, *Tribulus Terrestris*, *Urtica dioica*, *Adiantum capillus-veneris*, *Stigma maydis* (corn silk), and *Cucumis melo*, were investigated in a randomized, single-blinded, placebo-controlled clinical trial. It involved 54 participants (with renal stones less than or equal to 10 mm), 27 in the intervention and 27 in the placebo arms. The intervention group took standard treatment and 60 drops thrice daily for 1 month, and the control group took standard treatment plus placebo. An ultrasound scan was used to evaluated stone parameters in this study. The result showed significant stone expulsion and stone size reduction in the intervention group, and on the other parameters, only urine volume significantly differed [46]. The study concluded that the five-herb mixture is more effective in managing urinary lithiasis than the placebo.

Brardi et al., in a prospective randomized comparison study, investigated the effects of the combination of potassium citrate and *Agropyron repens* in renal stone treatment comparing it with potassium citrate alone. In an unblinded study, 50 participants were divided into two arms, the first 25 taking potassium citrate plus *Agropyron repens* combination and the other arm of 25 participants taking only potassium citrate. Both arms were advised to take the same diet and increased fluid intake in a 5-month follow-up period. There was a significant reduction in the total number of stones, stone diameter, and reduction in excreted uric acid in the combination arm compared with the potassium citrate-only group without a significant difference in citraturia, oxaluria, urinary calcium, and urinary pH. This study concluded that using the combination of couch grass and potassium citrate is safe and effective compared with potassium citrate alone to treat urinary stones [47].

Kristyantoro et al. investigated the effectiveness of the Renalof^®^ supplement, comparing it with Kalkurenal and placebo. Thirty patients with renal stones up to 2 cm measured by plain X-ray and ultrasound scans were recruited and divided into three arms: 9 took a placebo, 8 took Kalkurenal, and 13 were on Renalof^®^. After a month of intervention, the result showed a statistically insignificant decrease in all patients’ 24 h urine excretion of calcium and uric acid. Still, there was also a significant decrease in stone parameters in the Renalof^®^ arm. The study concluded that Renalof^®^ can be safely used as an adjunct treatment in urolithiasis [48].

Extracts from *Agropyron repens* (*Elymus repens*, *Elytrigia repens*, quack grass, couch grass), a worldwide infesting rhizomatous plant native to Europe and Central Asia [49], are the main constituents of the Renalof^®^ supplement along with mannitol and magnesium [50]. A double-blind, randomized, placebo-controlled, parallel-group phase III clinical trial was conducted to evaluate the safety and effectiveness of Renalof^®^ in the dissolution and expulsion of calcium-containing renal stones less than 10 mm in diameter. Renalof^®^ was taken as one tablet three times daily for 3 months in the treatment group with 52 patients, and the control arm had 58 patients taking a placebo three times daily. After 3 months, there was a 7.7% stone reduction and an 86.5% stone expulsion in the Renalof^®^ group compared with a 0% stone reduction in the placebo arm. The study concluded that Renalof^®^ is safe and effective in stone size reduction and expulsion [51]. Likewise, a phase II randomized, prospective, observational, single-blind study of 155 patients with urinary stones less than 10 mm that enrolled 120 patients in the treatment arm taking Renalof^®^ 325 mg twice daily for 3 months and 35 patients on placebo showed the safety and effectiveness of Renalof^®^. There was a 65% expulsion in the Renalof^®^ group compared with 11.4% in the placebo arm, a result in favor of Renalof^®^ [50].

Notably, a recent study pointed toward the safety, efficacy, and tolerability of a three-component herbal compound in a pediatric population with small stone fragments after endourological treatment. Patients were randomly divided into two groups and were followed up for 3 months. The drug proved an efficient ancillary treatment to prevent new stone growth and reduce residual fragments [52].

Renalof^®^ was evaluated in a recent placebo-controlled RCT that included 82 patients with predominantly calcium oxalate stones (Hounsfield units > 500) randomized to Renalof and placebo. Stone volume, surface, and location were evaluated by CT and analyzed using a dedicated computer software (InVesalius) both before and after the 3-month intervention period. From the 73 patients that were available for analysis (9 patients had passed their stones during the study period), those in the Renalof group had a statistically significant (approximately 25%) reduction in stone surface and stone volume at 3 months [53]. The study’s strong point was the use of a software to accurately measure stone surface and volume instead of maximum stone diameter (Table 1).

## 4. Phytotherapy: Something to Worry About or Much Ado About Nothing?

The increased use of herbal treatments has influenced the scientific community to get interested in their toxicity. Risk assessment tests have been developed for traditional herbal medicines [54]. “Safe” and “natural” are not synonyms; therefore, herbal medicines have to be treated as orthodox medicines to determine their mode of action, potential adverse reactions, contraindications, and interactions with other medicines [55]. Environmental pollutants and phytochemicals can affect the final product, and strides have been made in the authentication of herbal species, detection of harmful chemicals, toxicity mechanisms, pathway elucidation, and quantification of environmental pollutants in plants [56]. In a nutshell, despite the generalized use of phytotherapy in urolithiasis, there is a significant lack of high-level evidence published on these commonly utilized treatments [57]. Its conscious administration warrants attention to limit side effects, toxicity, and ineffective treatment [58]. Research evaluating the in vivo cytotoxicity and application of these regimens in animal cell models would be valuable and could provide further insights into their safety profile. The RCTs included in this narrative review also checked on the interventions’ adverse effect profile and were recorded. Drug interaction, though, was not determined.

## 5. Evidence from Systematic Reviews

A systematic review by Monti E et al. concluded that a herbal mixture induced stone clearance, size reduction, and stone clearance significantly better than a placebo. However, citrate was better than phytotherapy in decreasing stone size [59]. A recent systematic review found that phytotherapy can be used as an alternative treatment, but more research is still necessary to attain maximum treatment benefits [60]. Another systematic review of the anti-urolithic effects of medicinal plants on calcium oxalate stones in rats found favorable changes in the lithogenic factors and also a reduction in calcium oxalate crystal deposition in the kidneys despite having only a small percentage assessing the antioxidation and diuretic activities of these treatments [61].

## 6. Strengths, Limitations, and Future Directions

Our review provides a comprehensive overview of studies reporting on phytotherapy and herbal remedies in urolithiasis. It included various studies and provided an overall summary, interpreting and critiquing the results in a non-systematic manner [62,63]. This allowed the inclusion of varying studies to offer a readable, thoughtful, and practical synthesis of urolithiasis and its management through herbal medicine or phytotherapy in a broad spectrum [63,64]. This review is limited by its narrative nature. There is sometimes potential selection bias, selectiveness, and lack of exhaustiveness [63,64]. In this narrative review, extra care was taken to improve the thought process, stick to the purpose, and increase transparency in selecting the involved studies [63]. Selected RCTs, though, had heterogeneity in the herbs used, their chemical compositions, treatment period, radiology type used to determine lithiasis size and density, and dosages.

Urolithiasis has been high in developed countries and is also increasing in developing countries [65,66]. The use of herbal supplements for urolithiasis is increasing, and the long-term effects of these compounds are still uncharted territory. However, further studies are still required to determine doses, effective mixtures, mechanisms of action, drug interactions, and the efficacy of phytotherapy. This will assist in developing a predetermined concoction with doses and a treatment period, as is the case with current orthodox treatments for urolithiasis. Lastly, various preoperative assessment models have been developed to evaluate stone-free results following surgical intervention, which have significantly aided in the surgical planning process, particularly for Retrograde Intrarenal Surgery (RIRS) [67]. Similar nephrolithometric scoring systems might play a role in predicting outcomes after herbal therapy.

## 7. Conclusions

The prevalence of urolithiasis is high, and its management has significant economic implications in both developed and developing economies. Ancient treatment of urolithiasis was centered on herbal medicines, but conventional treatment modalities have overtaken phytotherapy along the way. Phytotherapy is bouncing back, and its use in urolithiasis is gradually increasing, albeit with limited clinical evidence to support its use. Thus, phytotherapy is increasingly involved in the management of urolithiasis in both prevention and treatment. Different plant extracts have undergone trials, some being mixtures and others being sole plant extracts, with varying results. Most studies show the effectiveness of phytotherapy in urolithiasis, but no exact modes of action have been established. Further studies are required to determine doses, modes of action, drug interactions, and duration of treatment depending on the phytotherapy used and the stone type.

## Figures and Tables

**Table 1:** Brief summary of studies reporting on the use of phytotherapeutic regimens for urolithiasis. RCT: randomized controlled trial; US: ultrasound; CT: computed tomography; NCCT: non-contrast computed tomography; vs.: versus; NS: not stated; *: weeks; ^†^: recurrent stone formers.

Authors, Year	Study Type	Regimen	Stone Size (mm)	Imaging Modality	Recruited/ Randomized	Maximum Period (Months)	Findings
Singh et al., 2011 [28]	RCT	*Sitivaraka* vs. potassium citrate	≥8 mm	US	21/23	6	-Stone size reduction
Pucci et al., 2018 [30]	RCT	*Phyllanthus Niruri* (Stone Breaker)	<10 mm	US CT	56	26 *	-Increased urinary excretion of magnesium and potassium
Cealan et al., 2019 [33]	RCT	*P. niruri* + Mg + VitB6	<15 mm	NCCT	48	3	-No change
Movaghati et al., 2019 [35]	RCT	*Nigella Sativa* (Black Seed)	≥5 mm	US	30/30	10 *	-Stone size reduction -Stone expulsion
Jalal et al., 2020 [36]	RCT	*Phaseolus vulgaris*	≤10 mm	US	60	6 *	-Stone size reduction -Stone expulsion
Erickson et al., 2011 [37]	RCT and Crossover	Cystone^®^	NS	CT	10 ^†^	12	Not effective
Palaniyamma and Jeyaraman 2017 [39]	RCT	Cystone^®^	5–12 mm	US	65	3	-Stone size reduction -Stone expulsion
Patki et al., 2010 [40]	RCT	Cystone^®^	5–10 mm	X ray US	26/26	6	-Stone size reduction -Stone expulsion
Patankar et al., 2020 [45]	RCT	Subap Plus	4–9 mm	NCCT	34/31	6	-Stone size reduction -Stone expulsion
Samandarian et al., 2023 [46]	RCT	5 herbal extracts	≤10 mm	US	27/27	4	-Stone size reduction -Stone expulsion
Brardi et al., 2012 [47]	RCT	Potassium citrate + Agropyron repens	NS	US	25/25	5	-Stone size reduction -Stone expulsion
Kristyantoro et al., 2012 [48]	RCT	Renalof vs. Kalkurenal vs. placebo	<20 mm	X ray US	13/8/9	1	-Stone size reduction -Stone expulsion
Chamorro et al., 2021 [50]	RCT	Renalof vs. placebo	<10 mm	US CT	120/35	3	Increased stone expulsion
Sánchez et al., 2012 [51]	RCT	Renalof vs. placebo	<10 mm	X ray US CT	52/58	3	-Stone size reduction -Stone expulsion
Caione et al., 2022 [52]	RCT	*Herniaria hirsuta* and *Peumus boldus*	Small stone fragments	NS	15/19	3	Stone expulsion post endourology
Sountoulides et al., 2024 [53]	RCT	Renalof vs. placebo	<2 cm	CT	82/82	3	-Stone surface and stone volume reduction

## Abbreviations

The following abbreviations are used in this manuscript:

PCNL	Percutaneous Nephrolithotomy
RIRS	Retrograde Intrarenal Surgery
URS	Ureteroscopy
SWL	Shockwave Lithotripsy
MET	Medical Expulsive Therapy
UTI	urinary tract infection
RCTs	randomized controlled trials
